# Does switching from coated colchicine to compressed colchicine improves treatment response in patients with familial Mediterranean fever?

**DOI:** 10.3325/cmj.2023.64.354

**Published:** 2023-10

**Authors:** Nimet Öner, Elif Çelikel, Zahide Ekici Tekin, Vildan Güngörer, Tuba Kurt, Müge Sezer, Nilüfer Tekgöz, Cüneyt Karagöl, Serkan Coşkun, Melike Mehveş Kaplan, Merve Cansu Polat, Banu Çelikel Acar

**Affiliations:** Ankara Bilkent City Hospital, Ankara, Turkey

## Abstract

**Aim:**

To evaluate the treatment response to compressed colchicine tablets in familial Mediterranean fever (FMF) patients with resistance or intolerance to coated colchicine. The secondary aim was to determine the demographic and clinical characteristics of responders to compressed colchicine.

**Methods:**

We retrospectively reviewed the medical records of 1574 pediatric patients with FMF treated at Ankara Bilkent City Hospital. Sixty-one patients did not respond to coated colchicine and were switched to compressed colchicine. In these patients, the number of attacks and the International Severity Score for FMF (ISSF) during the 6 months before and 3, 6, 9, 12, and 24 months after switching from coated colchicine to compressed colchicine were recorded.

**Results:**

Twelve of 61 patients (19.7%) who were switched to compressed colchicine due to intolerance responded to treatment. Of the 49/61 patients (80.3%) who were switched due to uncontrolled attacks and persistent subclinical inflammation, 25 responded to treatment. The frequency of attacks and ISSF decreased after switching. At the end of the two-year follow-up, 42 patients responded to compressed colchicine, and 19 patients received compressed colchicine plus interleukin-1-targeting drugs.

**Conclusions:**

Compressed colchicine was shown to be a useful treatment option before initiating biological agents in non-responders to coated colchicine, especially those with side effects.

Familial Mediterranean fever (FMF) is an autosomal recessive hereditary autoinflammatory disease characterized by recurrent attacks of fever, peritonitis, and pleuritis (1). Since 1972, colchicine has been used to prevent attacks and renal amyloidosis in FMF. Colchicine is an alkaloid extracted from the *Colchicum autumnale* plant (2). It inhibits neutrophil chemotaxis and activity in response to vascular injury and inflammasome signaling, decreases active interleukin-1β production, reduces neutrophil-platelet interaction and aggregation, and ultimately suppresses inflammation (3,4).

Colchicine resistance or intolerance is present in approximately 5%-10% of patients with FMF (1). While colchicine remains the mainstay of therapy in FMF, other therapeutic options, such as interleukin 1 (IL-1)-targeting drugs, are now used in patients with colchicine intolerance or resistance. Two of these drugs are anakinra, an IL-1 receptor antagonist, and canakinumab, a human-specific monoclonal anti-IL-1β antibody (5).

Although FMF is a prototypical autoinflammatory disease with the disease-associated gene identified 25 years ago, there are still unresolved issues, particularly in the management of colchicine intolerance or resistance. Several recent studies have shown that not all colchicine-containing preparations are equally effective (6-8). This has led to the use of an alternative colchicine preparation in FMF patients with colchicine intolerance or resistance before initiating IL-1-targeting drugs. Colchicine is available in the form of different pharmaceutical preparations, such as coated and compressed colchicine.

The effects of colchicine preparation switching on clinical outcomes have been scarcely investigated in the pediatric population. The aim of this study was to evaluate the treatment response to compressed colchicine tablets in patients with resistance or intolerance to coated colchicine used as first-line therapy in FMF in Turkey. We also aimed to determine the demographic and clinical characteristics of responders to compressed colchicine preparations.

## Patients and methods

### Patients and study design

This retrospective study involved patients who were diagnosed with FMF in the Pediatric Rheumatology Department of Ankara Bilkent City Hospital. Among 1574 patients with FMF who were followed-up since 2005 and who used coated colchicine, 68 patients who used compressed colchicine between 2018 and 2022 were included in the study. Patients who used compressed colchicine before 2018 were not included in the study due to missing data.

All patients met the Yalçınkaya-Özen criteria (9) for the diagnosis of FMF. The study enrolled patients who were <18 years of age, had received coated colchicine treatment for at least six months, and were switched to a compressed colchicine preparation due to colchicine resistance or intolerance. Exclusion criteria were the use of additional drugs due to comorbidities, non-compliance with colchicine use, and a follow-up period shorter than 24 months after switching to another colchicine preparation.

Demographic data, clinical and laboratory characteristics, colchicine doses, side effects, the International Severity Score for FMF (ISSF), and the results of genetic analysis of MediterraneanFeVer (*MEFV*) mutations were collected from patients’ medical records. Exons 2, 3, 5, and 10 in the *MEFV* gene were analyzed in patients with a clinical suspicion of FMF. The number of attacks and ISSF during the 6 months before and 3, 6, 9, 12, and 24 months after switching from coated colchicine to compressed colchicine were recorded.

### Definitions

Colchicine resistance was defined as ≥1 attack per month in compliant patients receiving the maximally tolerated dose for ≥6 months (10) or >3 attacks over 4-6 months (11) despite colchicine treatment. Additionally, it was defined as the persistence of subclinical inflammation during the attack-free period. Colchicine intolerance was defined as the inability to increase colchicine to an effective dose due to side effects.

The ISSF (12) is used to assess disease activity in FMF. ISSF is calculated based on the following criteria: 1) chronic sequelae, 2) organ dysfunction, 3) organ failure, 4) attack frequency, 5) elevation of acute phase reactants (erythrocyte sedimentation rate, C-reactive protein, serum amyloid A) during the remission period when at least two weeks or more has passed after the last attack whose frequency was lower than at least two per month, 6) involvement of two or more sites during an acute, patient-specific attack (eg, pericarditis, pleuritis, peritonitis), 7) more than two types of attacks during the course of the disease, 8) attack duration, and 9) leg pain upon exercising. The frequency of attacks is awarded 0-2 points, other parameters are awarded 0-1 points, and the maximum score is 10 points. The disease is classified into mild (<3 points), moderate (3-5 points), and severe (>5 points).

Liver enzymes were considered to be elevated if there was at least a 3-fold elevation in aspartate aminotransferase or alanine aminotransferase. Diarrhea was defined as at least two liquid stools per day or at least two liquid stools per week despite a minimum appropriate treatment dose.

### Treatment and doses

According to the EULAR (10), the recommended colchicine dose is ≤0.5 mg/d for children under 5 years of age (≤0.6 mg/d if the tablets contain 0.6 mg), 0.5-1.0 mg/d for children aged 5-10 years (1.2 mg/d if the tablets contain 0.6 mg), and 1.0-1.5 mg/d (1.8 mg/d if tablets contain 0.6 mg) for children aged >10 years and adults.

Coated colchicine preparations available in Turkey are Colchicum-Dispert® (Recordati, Istanbul, Turkey) and Kolsin® (İbrahim Etem Ulagay, Istanbul, Turkey), which contain 0.5 mg of colchicine per tablet.

Compressed colchicine preparations, Colchicine Opocalcium® (Mayoly Spindler, Chatou, France), ColchicinaLirca® (Acarpia, Milano, Italy), and Colchicine Seid® (Seid Lab, Barcelona, Spain) are used with the approval of the Turkish Pharmacists Association in patients who are intolerant or unresponsive to coated colchicine. These preparations contain 1 mg of colchicine per tablet. The recommended IL-1-targeting drugs doses are 1-2 mg/kg/d for anakinra and 2-4 mg/kg/mo for canakinumab.

This study complied with the Declaration of Helsinki and was approved by the Ethics Committee of the Ankara City Hospital (E2-21-1172).

### Statistical analysis

The normality of the distribution was assessed with the Kolmogorov-Smirnov and Shapiro-Wilk tests. Data are expressed as the mean ± standard deviation (SD). The analysis was conducted with SPSS, version 26.0 (IBM Corp., Armonk, NY, USA).

## Results

**Figure 1:**
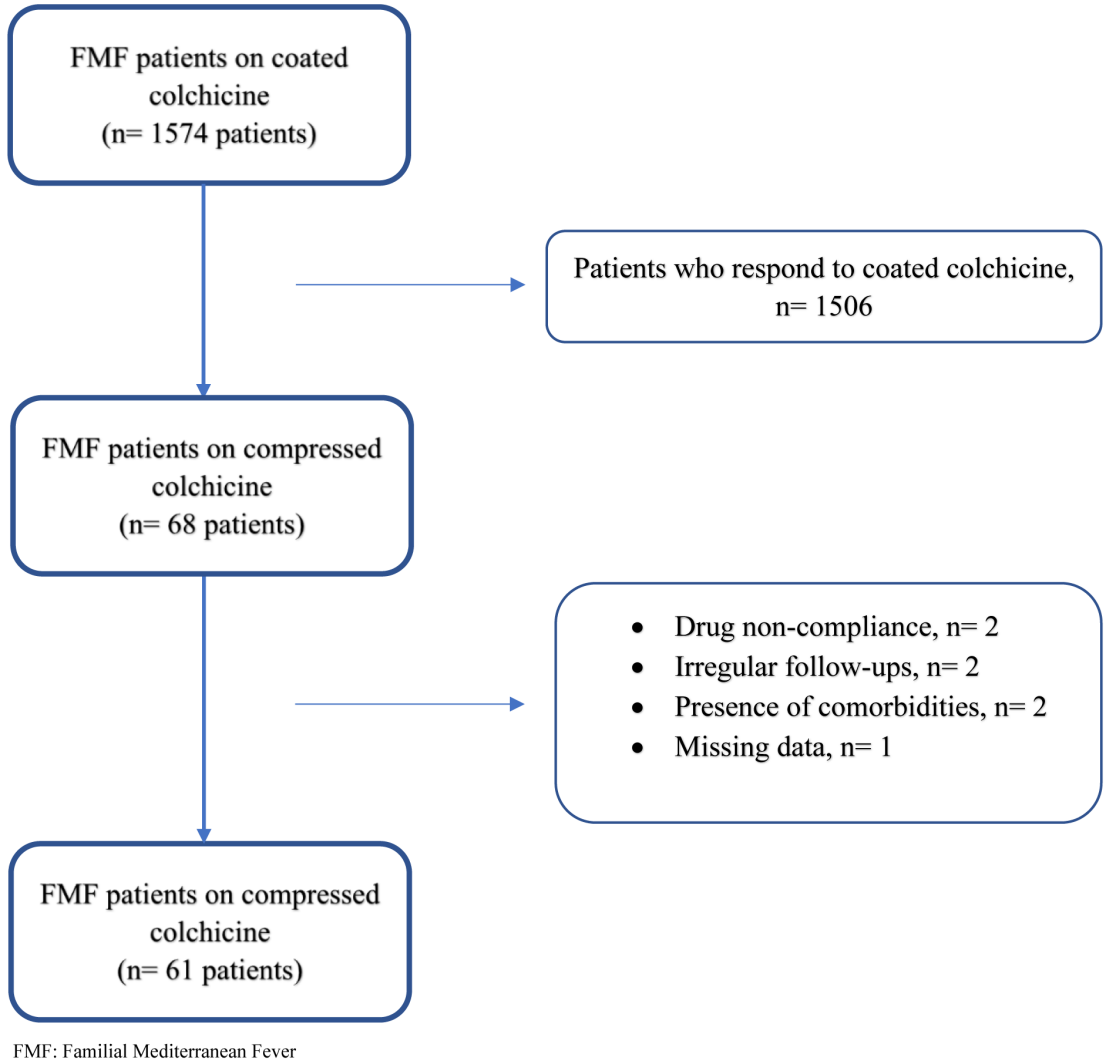
Study inclusion flowchart.

This study enrolled 61 patients who were switched from coated colchicine to compressed colchicine due to resistance or intolerance (Figure 1). Demographic features, clinical findings, and genetic test results are presented in Table 1 and Table 2. The most common mutation observed in the *MEFV* gene analysis was homozygous M694V mutation (35 patients; 57.3%). In 5 patients, *MEFV* mutation analysis results were negative. These patients underwent further genetic testing for other autoinflammatory diseases. The results of these genetic analyses were also negative.

**Table 1:** Patients’ demographic and genetic characteristics

Characteristics	Compressed colchicine responders; n = 42	Compressed colchicine non-responders; n = 19	Total; n = 61
Sex, n (%)			
Female	25 (59.5)	11 (57.9)	36 (59)
Male	17 (40.5)	8 (42.1)	25 (41)
Mean age of symptom onset, months ± SD	42 ± 28	18 ± 11	34 ± 22
Mean age at diagnosis, years ± SD	8.7 ± 5.3	7.8 ± 4.7	8.4 ± 5.1
Mean age at compressed colchicine onset, years ± SD*	12.4 ± 3.9	10.6 ± 2.6	11.8 ± 3.5
Results of genetic analysis, n (%)			
**Exon 10/Exon 10**			
M694V/M694V	24 (57.1)	11 (57.9)	35 (57.4)
M694V/M680I	6 (14.3)	1 (5.26)	7 (11.5)
M680I/M680I	0	1 (5.26)	1 (1.64)
M680I/V726A	1 (2.4)	0	1 (1.64)
**Exon 10/Exon 2**			
M694V/E148Q	2 (4.8)	1 (5.26)	3 (4.9)
**Exon 10/ -**			
M694V/-	4 (9.5)	2 (10.53)	6 (9.8)
V726A/-	1 (2.4)	0	1 (1.64)
R761H/-	1 (2.4)	0	1 (1.64)
**Exon 2/ -**			
E148Q/-	0	1 (5.26)	1 (1.64)
**No mutation**	3 (7.1)	2 (10.53)	5 (8.2)

*SD - standard deviation.

**Table 2:** Patients’ clinical characteristics

Characteristics, n (%)	Compressed colchicine responders; n = 42	Compressed colchicine non-responders; n = 19	Total patients; n = 61
Abdominal pain	36 (85.7)	16 (84.2)	52 (85.2)
Fever	31 (73.8)	14 (73.7)	45 (73.8)
Arthralgia	18 (42.9)	9 (47.4)	27 (44.3)
Erysipelas-like erythema	9 (21.4)	5 (26.3)	14 (23)
Arthritis	1 (2.4)	10 (52.6)	11 (18)
Chest pain	6 (14.3)	3 (15.8)	9 (14.8)
Prolonged febrile myalgia	3 (7.1)	2 (10.5)	5 (8.2)

The mean erythrocyte sedimentation rate, C-reactive protein, serum amyloid A values of the patients during their attack-free periods were 5.3 ± 2.7 mm/h, 6.1 ± 3.5 mg/L, and 5.2 ± 2.1 mg/L, respectively. 11/61 patients had elevated acute phase reactants in their attack-free periods.

### Follow-up after switching from coated colchicine to compressed colchicine

The reasons for switching to compressed colchicine were uncontrolled attacks and persistence of subclinical inflammation in 49 patients (80.3%) and intolerance in 12 patients (19.7%; 3 patients had increased liver function test results, 2 patients had lymphopenia, and 7 patients had gastrointestinal side effects).

**Figure 2:**
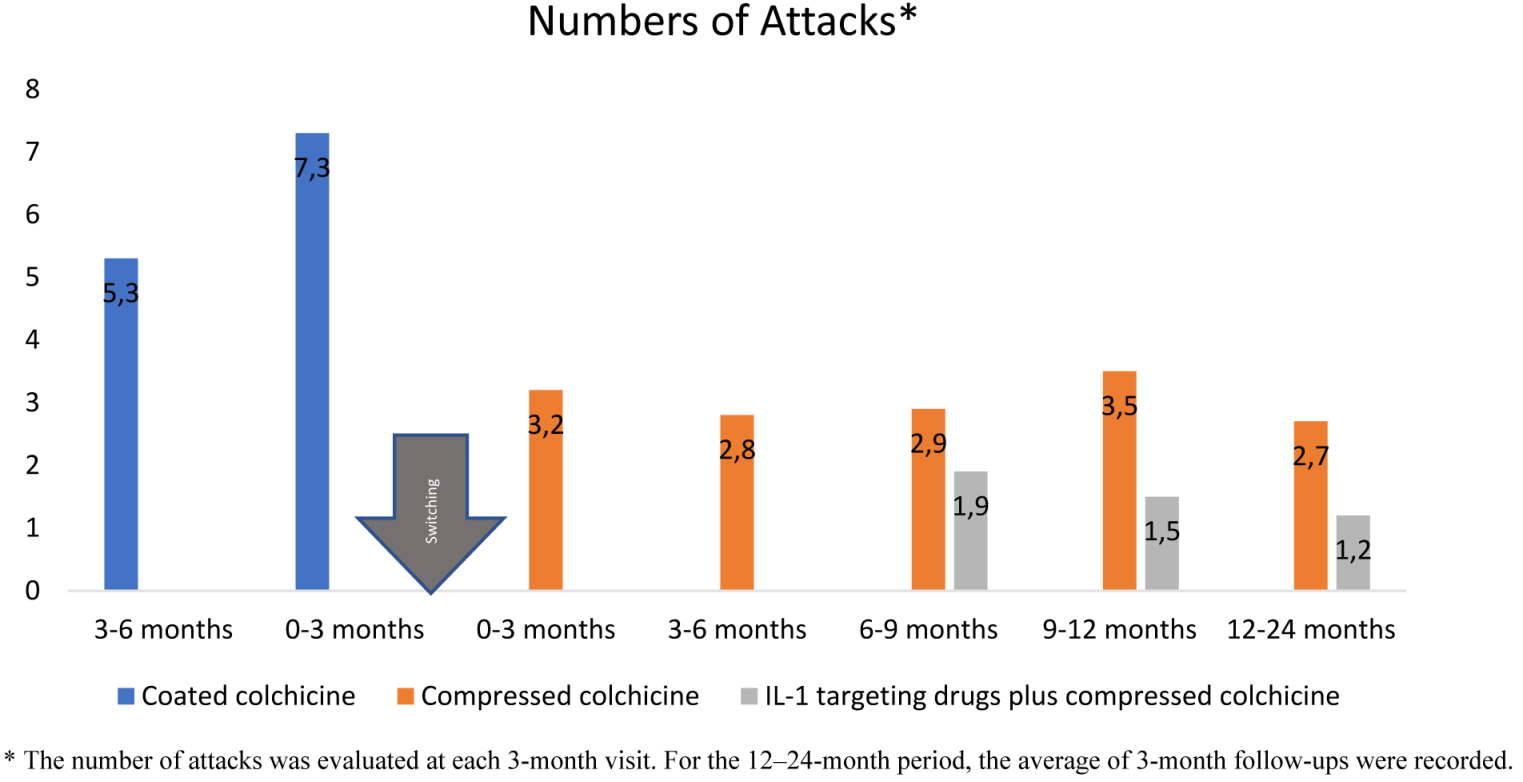
The number of attacks in patients switched from coated colchicine to compressed colchicine. The number of attacks was evaluated at each 3-month visit. For the 12-24-month period, the average of 3-month follow-ups was recorded.

**Figure 3:**
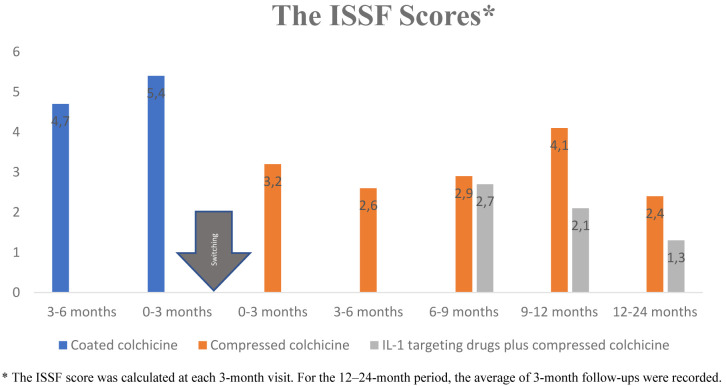
The International Severity Score for familial Mediterranean fever (ISSF) of patients switched from coated colchicine to compressed colchicine. The ISSF score was calculated at each 3-month visit. For the 12-24-month period, the average of 3-month follow-ups was recorded.

The number of attacks at 3 and 6 months before switching and at 3 and 6 months after switching is shown in Figure 2. The frequency of attacks decreased at 3 and 6 months after switching. The mean ISSF value in the last 3 months before switching was 5.4 ± 2.3, while after switching the lowest value was 1.3 ± 0.7, observed between 12 and 24 months (Figure 3). Twelve patients who were switched due to intolerance responded to compressed colchicine and did not require additional treatment.

### The follow-up of non-responders to compressed colchicine, and addition of IL-1-targeting drugs

Forty-nine of 61 patients were switched to compressed colchicine due to indications other than colchicine intolerance, including uncontrolled attacks and subclinical inflammation. Of the 49 patients, 25 responded to compressed colchicine treatment. The demographic characteristics, clinical characteristics, and *MEFV* gene analysis results of the patients who responded to compressed colchicine and those who did not are shown in Tables 1 and 2. The non-respondents were diagnosed with colchicine-resistant FMF, and IL-1-targeting drugs were concomitantly added to their treatment (anakinra in 14 patients and canakinumab in 10 patients).

**Figure 4:**
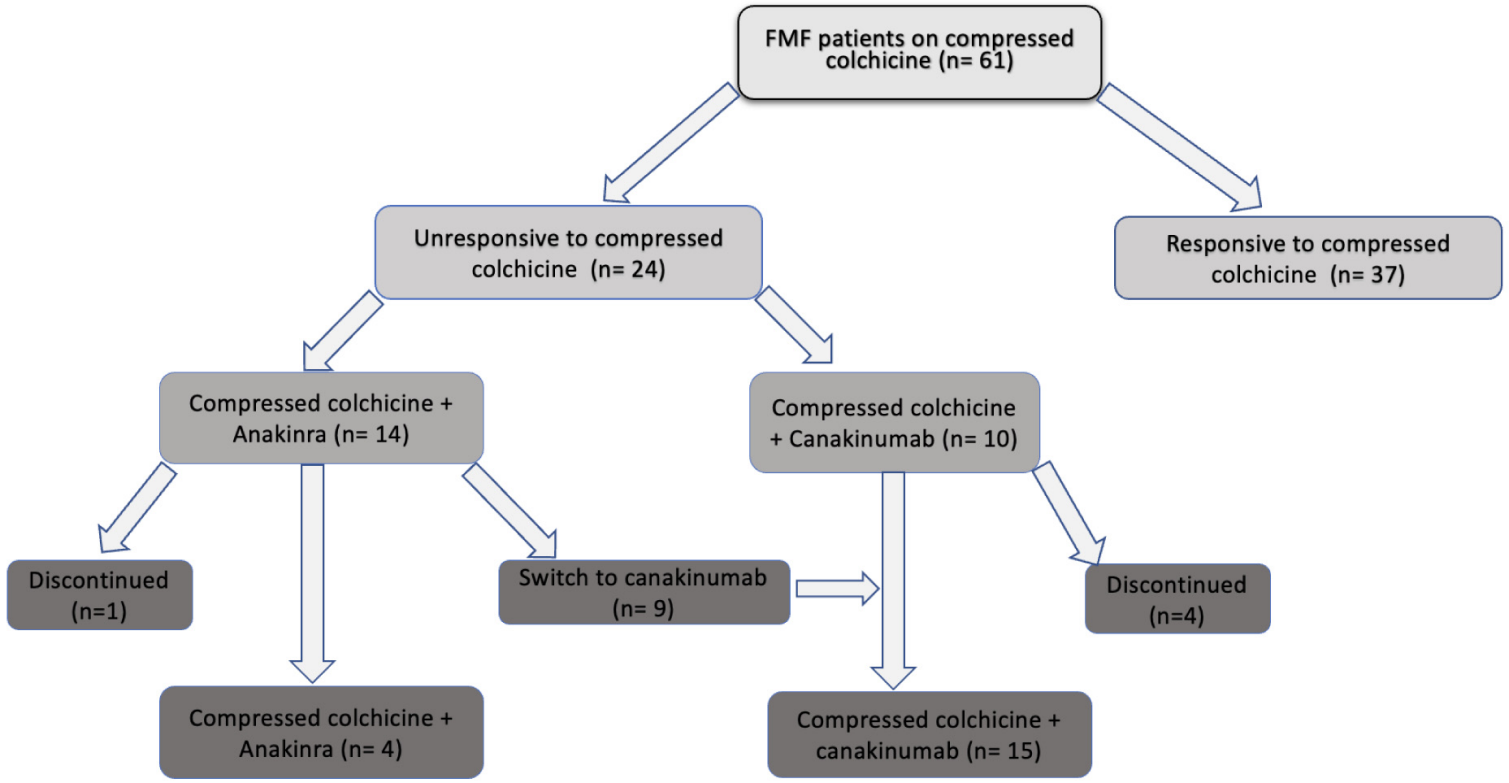
The management of patients treated with interleukin 1-targeting drugs.

The management of patients using IL-1-targeting drugs is outlined in Figure 4. The median treatment duration was 6.7 months (range 4-14 months) for anakinra and 9.1 months (range 6-16 months) for canakinumab.

The number of attacks in patients who received compressed colchicine alone and in non-responders to compressed colchicine who were additionally given IL-1-targeting drugs is shown in Figure 2. After the addition of IL-1-targeting drugs, the number of attacks among the patients decreased in all months.

There were no differences in terms of sex, age at diagnosis, clinical characteristics (except for arthritis), or *MEFV* mutations between treatment responders and those who did not respond to colchicine and were additionally given IL-1-targeting drugs (Tables 1 and Table 2). Arthritis was more common in patients who were additionally administered IL-1-targeting drugs. Arthritis occurred in 1 of 42 treatment responders and in 10 of 11 patients who additionally received IL-1-targeting drugs. The rate of colchicine resistance was higher in patients who had symptoms onset at an earlier age.

At the end of the two-year follow-up, 42/61 were using compressed colchicine alone, 4/61 were using anakinra in addition to compressed colchicine, and 15/41 were using canakinumab in addition to compressed colchicine. None of the patients had secondary amyloidosis.

## Discussion

This study showed that 25 of the 49 non-responders to coated colchicine and 12 patients with colchicine intolerance experienced significant improvement after switching to compressed colchicine.

Colchicine is an alkaloid that inhibits many cellular functions, including microtubule assembly, cell adhesion, and inflammasome activation (13,14). It is mainly metabolized in the liver via demethylation by the cytochrome P450 system (15). Since it is the main treatment for FMF, it is available in both oral and intravenous (IV) forms. The accepted pharmaceutical form is the oral tablet form, and the IV form has often been tested in clinical trials. Grossman et al reported that the long-term use of the IV form was effective and safe in patients who did not respond to oral (tablet, solution) colchicine (16). Oral colchicine tablets are available as coated (film-coated, sugar-coated) or compressed tablets, and they are produced in many countries in different preparations.

A few studies have investigated the effects of colchicine preparation switching on clinical outcomes (6-8). Emmungil et al reported a significant decrease in attack frequency and activity scores (AIDAI and PRAS) in 50 adult patients who were switched from coated colchicine to compressed colchicine. The authors attributed these favorable outcomes to differences in the pharmacokinetic properties of different colchicine preparations (6). Baglan et al showed a decreased frequency and duration of attacks in 35 pediatric patients after switching from domestically produced colchicine to foreign-produced compressed colchicine. In their study, the mean frequency of attacks decreased from 9.50 ± 4.46 before to 1.85 ± 1.41 a year after treatment with compressed colchicine, and inflammatory marker levels significantly decreased (7). Türkuçar et al found that the frequency of attacks and gastronintestinal side effects decreased after switching from coated colchicine to compressed colchicine preparations in 29 pediatric patients. FMF severity scores and Bristol stool chart questionnaire scores improved significantly after the switch. They concluded that switching colchicine preparations may favorably affect both attack frequency and intolerance (8). Similar to previous studies, in this study switching to compressed colchicine led to favorable outcomes in non-responders to coated colchicine.

In our study, the desired response was not obtained in 24 of the 49 patients who were switched from coated colchicine to compressed colchicine. These 24 patients were considered colchicine-resistant, and received additional IL-1-targeting treatment. Increased IL-1 production is associated with mutations in the pyrin protein. Inhibiting the activity of IL-1 with IL-1-targeting drugs is therefore a logical step in controlling inflammation in colchicine-resistant FMF patients. Despite a favorable response to anakinra, 9/14 patients were switched to canakinumab due to non-compliance with daily injections and other reasons. Previous studies (17-19) suggest that canakinumab can be used in patients who do not respond to anakinra, although it is not a recommended agent in patients who do not respond to colchicine.

In the present study, the frequencies of attacks and ISSF values decreased after the addition of IL-1-targeting drugs. Additionally, similar to previous studies (20-22), the colchicine-resistant group had younger age at symptom onset and a higher frequency of arthritis compared with colchicine responders. Furthermore, colchicine-resistant patients were predominantly M694V homozygotes: 73.9% had M694V homozygous mutation and 78.9% had at least one M964V mutation. All patients except one had at least one exon-10 mutation. In view of these findings, patients using coated or compressed colchicine with an early onset of symptoms, a higher frequency of arthritis, higher ISSF values, and exon 10 mutations should be followed-up more closely.

The limitations of our study are its retrospective, single-center design and a relatively small sample size. Another limitation was the lack of plasma or leukocyte colchicine measurements. Despite these limitations, the study showed compressed colchicine to be a useful treatment option before initiating biological agents in non-respondents to coated colchicine. Switching to compressed colchicine should be considered, especially in patients with side effects to coated colchicine.
